# Association between Cardiovascular Disease Risk Factors and Cardiorespiratory Fitness in Firefighters: A Systematic Review and Meta-Analysis

**DOI:** 10.3390/ijerph20042816

**Published:** 2023-02-05

**Authors:** Jaron Ras, Andre P. Kengne, Denise L. Smith, Elpidoforos S. Soteriades, Lloyd Leach

**Affiliations:** 1 Department of Sport, Recreation and Exercise Science, Faculty of Community and Health Sciences, University of the Western Cape, Cape Town 7535, South Africa; 2 Non-Communicable Diseases Research Unit, South African Medical Research Council, Cape Town 7505, South Africa; 3 Health and Human Physiological Sciences, Skidmore College, Saratoga Springs, NY 12866, USA; 4 Healthcare Management Program, School of Economics and Management, Open University of Cyprus, Nicosia 2220, Cyprus; 5 Department of Environmental Health, Environmental and Occupational Medicine and Epidemiology (EOME), Harvard T.H. Chan School of Public Health, Boston, MA 02115, USA

**Keywords:** firefighters, cardiovascular disease risk factors, cardiorespiratory fitness, aging, obesity, hypertension, dyslipidemia, diabetes, systematic review

## Abstract

Approximately 45% of on-duty related mortalities were due to sudden cardiac death, with many of these fatalities related to cardiovascular disease and overexertion, while performing emergency duties. Therefore, the aim of this systematic review was to determine the association between cardiovascular disease risk factors and cardiorespiratory fitness in firefighters. A literature search of PubMed, SCOPUS, Web of Science, Embase, EBSCOHost, and ScienceDirect was conducted; the Rayyan^®^ intelligent systematic review tool was used to screen and select studies for inclusion. The appraisal tool for cross-sectional studies and the Critical Appraisal Skills Programme toolkit were used for methodological assessment of included studies. Data were analyzed using the Review Manager 5.3 and MedCalc^®^ statistical softwares to determine the effects of obesity (Z = 10.29, *p* < 0.001) and aging (Z = 4.72, *p* < 0.001) on cardiorespiratory fitness. Furthermore, there was a significant effect for cardiorespiratory fitness level on systolic blood pressure (Z = 5.94, *p* < 0.001), diastolic blood pressure (Z = 2.45, *p* < 0.001), total cholesterol levels (Z = 3.80, *p* < 0.001), low-density lipoprotein cholesterol (Z = 4.44, *p* < 0.001), triglycerides (Z = 3.76, *p* < 0.001) and blood glucose concentration (Z = 4.78, *p* < 0.001). Cardiovascular disease risk factors and cardiorespiratory fitness were significantly and inversely associated in firefighters. Fire service departments should adopt behavioral intervention strategies to maintain optimum cardiovascular disease risk factor profiles and cardiorespiratory fitness among firefighters to ensure their occupational well-being.

## 1. Introduction

Firefighting is an extremely hazardous occupation, where firefighters not only are required to contend with severe temperatures but are also required to perform emergency rescues and are routinely exposed to hazardous chemicals and fumes [1,2,3,4]. The hazardous nature of the profession requires firefighters to wear personal protective equipment (PPE), and in some departments, PPE can weigh up to 29.3 kg [5], which places significant strain on firefighters [6,7,8]. In addition, many firefighters have been reported to have underlying cardiovascular disease (CVD) risk factors, which significantly predispose them to cardiovascular events while on duty [9,10,11,12]. This translates into an excessively high mortality rate of nearly 50% related to on-duty sudden cardiac death (SCD) [1,2,3,13]. This is largely due to the physically demanding nature of their occupation, which requires firefighters to sustain a high level of physical intensity for prolonged periods, particularly when involved with fire suppression [2,3]. If underlying CVD risk factors are present, this predisposes firefighters to cardiac incidents while on duty [1,4]. Therefore, firefighters are expected to maintain optimum cardiovascular conditioning to reduce the likelihood of duty-related morbidity and mortality [2,3,14,15].

The most prevalent CVD risk factors among firefighters include obesity ranging between 14% and 60% [16,17,18,19], hypertension between 10% and 44% [17,20,21,22,23], cigarette smoking ranging between 11% and 39% [24,25,26,27,28,29,30], dyslipidemia between 20% and 56.5% [17,27,31,32,33], and physical inactivity between 14.7% and 70% [10,34,35,36,37,38,39]. These risk factors have been reported to consistently lower cardiorespiratory fitness in firefighters [30,35,40,41,42], primarily due to reducing vascular elasticity, reducing preload, and increasing afterload, and subsequently, reducing stroke volume and oxygen transportation to working muscles [43,44,45,46,47]. Firefighters, by maintaining their cardiorespiratory fitness through regular physical activity, can reduce their likelihood of CVD events, especially as they age [35,48,49]. Moreover, maintaining a healthy diet can assist in reducing the likelihood of CVD [50,51,52,53]. Previous systematic reviews have focused on the impact of firefighting tasks and the physiological responses while completing those tasks [54], and the physiological responses of firefighters that wear PPE and self-contained breathing apparatus gear [8]. Two studies have reported that significant physiological responses occurred while firefighters performed occupational tasks and especially while wearing full PPE and breathing apparatuses [8,54]. We are not aware of systematic reviews conducted on the effect of CVD risk factors on cardiorespiratory fitness in firefighters. The relatively low number of studies published on the effect of CVD risk factors on cardiorespiratory fitness is a concern, given the high number of CVD-related fatalities in firefighters [2,4,13,55]. Crucial new knowledge on the effect of CVD risk factors and which risk factors have the most significant effect on cardiorespiratory fitness is needed to assist in the formulation of policies to assist in maintaining firefighters’ cardiovascular health and fitness.

Therefore, this systematic review aimed at determining the association between CVD risk factors and cardiorespiratory fitness in firefighters. The research question guiding this study was: What is the association between CVD risk factors and cardiorespiratory fitness in firefighters?

## 2. Materials and Methods

**Figure 1:**
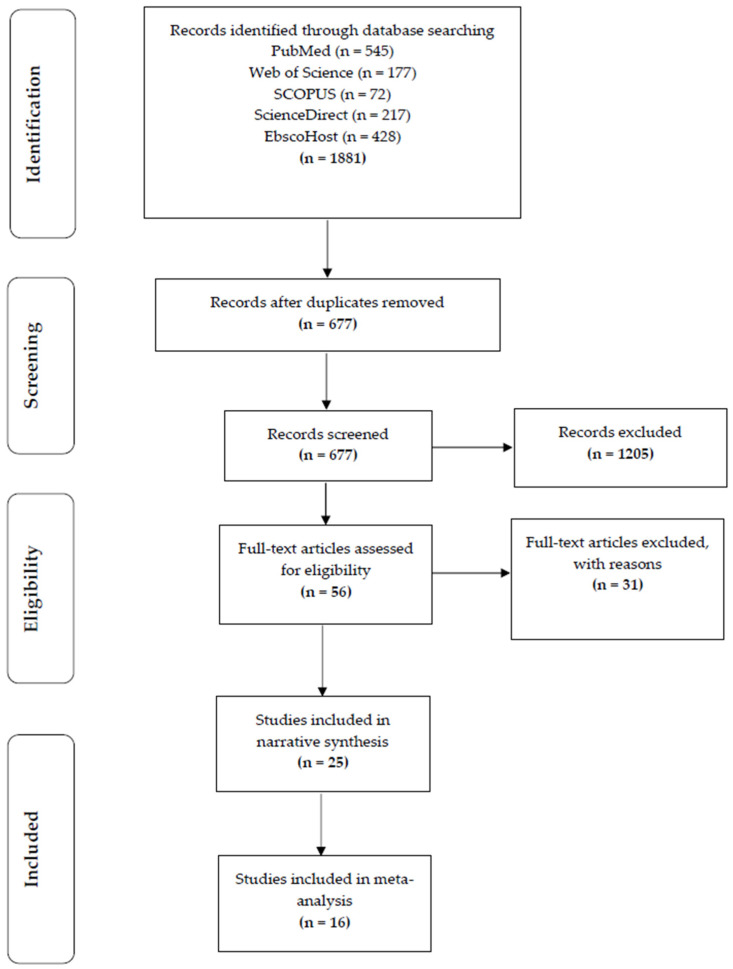
Flow chart of study selection in narrative review [62].

Each phase of the screening procedure was completed in accordance with the PRISMA standards for systematic reviews, which are illustrated in a flow diagram [56] (Figure 1). The guidelines for Meta-analysis of Observational Studies in Epidemiology (MOOSE) and Quality of Reporting of Meta-analysis (QUOROM) were utilized to complete the methodology of the current review [56,57].

### 2.1. Summary of Methods

The exposures assessed included CVD risk factors in relation to cardiorespiratory fitness in firefighters. There were no limitations to the publication year when considering studies for this review.

The inclusion criteria were as follows:

(i) Studies that included full-time, part-time, and volunteer adult male and female firefighters between the ages of 18 and 65 years;
(ii) Cross-sectional, observational, and experimental (intervention) study designs;
(iii) Studies that investigated the association between CVD risk factors or health metrics and cardiorespiratory fitness in firefighters;
(iv) Studies that were available in full text or that could be acquired through a request from the authors.

The exclusion criteria were as follows:

(i) Studies that failed to mention exposure and outcome measures, such as cardiorespiratory fitness and risk factors for cardiovascular disease;
(ii) Various forms of reviews, such as systematic reviews, literature reviews, scoping reviews, etc.;
(iii) Non-English language articles.

The comprehensive systematic review protocol can be found on PROPERO (CRD42022330510).

### 2.2. Study Selection, Screening, and Data Management

To find all pertinent papers, the two primary reviewers (J.R. and R.N.) conducted a thorough literature search of Web of Science, SCOPUS, PubMed/Medline, ScienceDirect, and EBSCOHost, using a combination of search terms (Supplementary File S1). Search outputs were compiled into the Zotero™ reference software, where duplicates were then removed. The remaining entries were imported into Rayyan Systems Inc. [58] where the titles and abstracts were screened for eligibility, using the categories of included, excluded, and unsure. Reasons for exclusion were provided as comments for each excluded study. The two principal reviewers used an initial rudimentary data extraction form (Supplementary File S2) to obtain the primary characteristics of each study, which comprised general study information, including authors and affiliations, publication date, study title, design, and country, as well as the exposure evaluated and outcome measures. Then, using a thorough data extraction form, study parameters were retrieved, including the sampling strategy, sample size, and participant’s information that included age, height, weight, body mass index (BMI), percentage of body fat, and maximum oxygen consumption (VO_2_max) (Supplementary File S3). Lastly, the details of the main exposures and outcomes for the current review were extracted and included CVD risk factors and cardiorespiratory fitness measures. Studies that qualified for the meta-analysis were entered into the Review Manager 5.3 [59] (The Cochrane Collaboration, London, UK) and MedCalc^®^ statistical software Ltd. (Ostend, Belgium, version 20.104).

### 2.3. Critical Appraisal of Included Studies

The methodological evaluation of the included studies was carried out using the appraisal tool for cross-sectional studies (AXIS checklist) (Table S1, Supplementary File S4) [60] and The Critical Appraisal Skills Programme (CASP) toolkit (Middle Way, Oxford, UK) (Table S2 Supplementary File S4) ((https://casp-uk.net/casp-tools-checklists/) (accessed on 1 September 2021)). For evaluating the quality of cross-sectional studies, it has been demonstrated that the CASP toolkit (Middle Way, Oxford, UK) and the AXIS toolkit are relevant and dependable resources [60,61]. According to the AXIS checklist, each study that was given a score, between 15 and 17 points was considered to be “good” quality and a score from 18 to 19 points was considered to be “high” quality. For the CASP toolkit, all studies were considered to be high quality, as all boxes were ticked.

### 2.4. Study Selection

The database searches yielded a total of 1881 entries (Figure 1). Of these, 1205 duplicates were removed. Of the remaining 677 studies screened via titles and abstracts, 56 studies met the inclusion criteria, and full texts were screened for final inclusion in the review. Reasons for exclusion included not investigating either the exploratory or outcome variables; studies that were qualitative in design, the main focus of the manuscript not being aligned with the current study, studies that were reviews, and studies that were not available in full-text. Following the full-text screening, 31 papers, in total, were eliminated, leaving 25 studies eligible to move forward with the data extraction and narrative synthesis. The reasons for exclusion included the association between the exploratory variable and the outcome variable not being analyzed. From this, 16 studies were suitable for inclusion in the meta-analysis after data extraction. Nine studies were excluded from the meta-analysis due to the data not being suitable for the analysis. The reasons for exclusion were, firstly, that studies did not include the means and standard deviation for both the CVD risk factors and the cardiorespiratory fitness measures. Secondly, there were no comparisons made between healthy firefighters and those with CVD risk factors and firefighters with good or poor cardiorespiratory fitness.

### 2.5. Data Analysis

#### 2.5.1. Assessment of Overall Effect Size

The data were imported and analyzed using Review Manager 5.3 [59,63,64,65]. The outcome measure (cardiorespiratory fitness) was analyzed as a continuous variable and dichotomous variable. The mean difference (MD), with a 95% confidence interval (CI) of estimation was used as effect estimates of the association between cardiovascular disease risk factors and cardiorespiratory fitness in firefighters [64]. For cardiorespiratory fitness measures that were dichotomous, the measure of 12 METS or greater was used to classify firefighters as being fit, and less than 12 METS as unfit. A meta-analysis of correlations was conducted using the MedCalc^®^ statistical software Ltd. (Ostend, Belgium, version 20.104). Using the inverse variance approach, the R values from the pooled studies were grouped based on correlation coefficients of similar exposures and outcomes and aggregated into a single, exemplary effect estimate [66]. The following formula was used to convert the original R values to a general test measure using Fisher’s “R” to “Z” transformation [66]:

(1) $$Z_{\mathrm{ri}}=\frac{1}{2}\ln\left(\frac{1+ri}{1-ri}\right)\;\;s\frac{2}{z}=\frac{1}{n-3}$$

To ascertain the correlation between the exposure and the outcome variables, the Fisher’s Z values from the original studies were pooled using the random effect model [67].

Interpretation of correlation coefficient strength [67]:

Very high correlation, from 0.90 to 1.00 (−0.90 to −1.00);
High correlation, from 0.70 to 0.90 (−0.70 to −0.90);
Moderate correlation, from 0.50 to 0.70 (−0.50 to −0.70);
Low correlation, from 0.30 to 0.50 (−0.30 to −0.50);
Negligible correlation, from 0.00 to 0.30 (−0.00 to −0.30).

#### 2.5.2. Classification of Cardiovascular Disease Risk Factors and Cardiorespiratory Fitness

Across studies, similar criteria were used to classify CVD risk factors. Age, as a risk factor, was classified as an age ≥45 years for males and ≥55 years for females. Obesity was classified as a body mass index (BMI) ≥30 kg·m^−2^. Hypertension was classified as a systolic blood pressure ≥140 mm Hg, or a diastolic blood pressure ≥90 mm Hg, or having been previously diagnosed with hypertension by a medical professional. Cigarette smoking was classified as being a current cigarette smoker or having quit within 6 months. Physical inactivity was classified as not exercising at a moderate intensity for at least 30 min, for at least three times a week. Dyslipidemia was classified as a total cholesterol concentration ≥5.18 mmol·L and a low-density lipoprotein ≥3.34 mmol·L or having been diagnosed by a medical professional. Diabetes was classified as a fasting blood glucose concentration ≥7.0 mmol·L or having a diagnosis by a medical professional. Firefighters were classified as being fit by meeting the minimum required cardiorespiratory fitness levels of 12 metabolic equivalents (METS), which is an oxygen consumption (VO_2_) of 42 mL·kg·min, that has been accepted as the minimum required cardiorespiratory fitness level needed for the profession. Firefighters that did not meet the 12 METS cut-off were categorized as not meeting the minimum required cardiorespiratory fitness level. Studies using the 12 METs cut-off were categorized as dichotomous variables and the CVD risk factors were continuous. Studies using relative VO_2_ were used as a continuous variable and CVD risk factors were used as dichotomous variables.

#### 2.5.3. Assessment of Heterogeneity and Publication Bias

The I^2^ and chi-square tests were employed as the two techniques to determine the degree of heterogeneity amongst the included studies [60]. These techniques have been used in previous meta-analyses to evaluate the effect of heterogeneity of the meta-analysis [60].

The following criteria were used to interpret I^2^ statistics:

(i) From 0% to 30%, indicated heterogeneity may not be important;
(ii) From 31% to 60%, indicated that there was moderate heterogeneity;
(iii) From 61% to 80%, indicated that there was substantial heterogeneity;
(iv) From 81% to 100%, indicated that there was considerable heterogeneity.

#### 2.5.4. Risk of Bias

The Begg’s test and Egger’s test were run using the Statistical Package for the Social Sciences (SPSS^®^, Chicago, IL, USA) version 28 to evaluate the risk of bias in the studies included in the meta-analysis.

#### 2.5.5. Subgroup Analysis and Investigation of Heterogeneity

To account for the presence of heterogeneity, a subgroup analysis was performed to explore the possible sources of the heterogeneity within studies [61]. The subgroup analysis was conducted on studies that used either a treadmill testing protocol, bicycle ergometer protocol, field tests, non-exercise measures, and direct VO_2max_ estimation using gas analysis.

## 3. Results

### 3.1. Study Characteristics

The included studies encompassed 21 cross-sectional studies, three cohort studies, and one case-controlled study, conducted between 1991 and 2021, and included a total of 7822 firefighters. Studies were conducted on fire departments located in various regions globally, where multiple variations of cardiorespiratory fitness tests were used to estimate cardiorespiratory fitness. A summary of the included studies is found in Table 1. The detailed critical appraisal of the studies may be found in Supplementary File S4.

### 3.2. The Association between Aging and Cardiorespiratory Fitness in Firefighters

**Figure 2:**
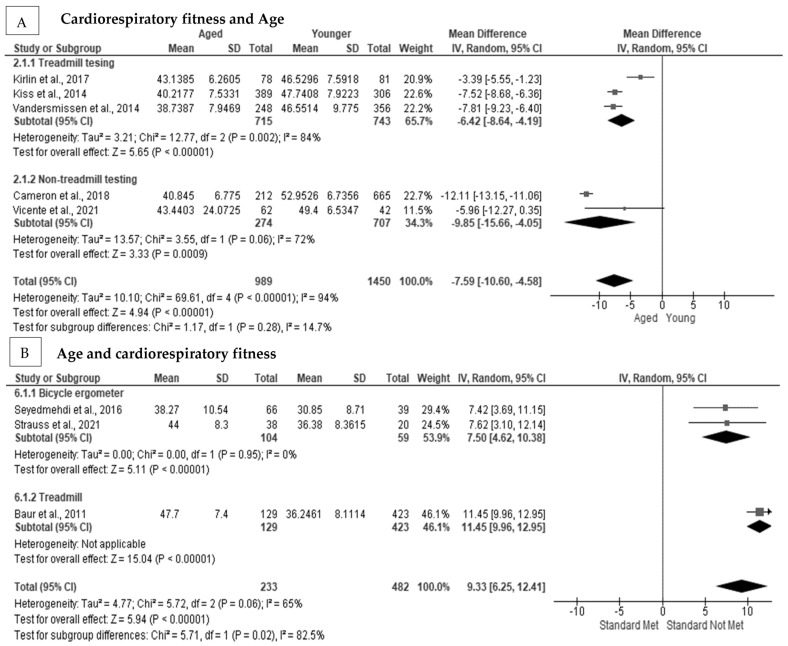
The association between age and cardiorespiratory fitness in firefighters: (**A**) The association between age and cardiorespiratory fitness with subgroup analysis [71,74,75,83,84]; (**B**) the association between cardiorespiratory fitness and age with subgroup analysis [30,40,69].

Aged firefighters had a significantly worse cardiorespiratory fitness level as compared with younger firefighters [71,72,73,74,75,83,84]. In addition, aged firefighters were less likely to meet the minimum cardiorespiratory requirements for firefighting. In Figure 2A, age had a significant negative pooled random effect on cardiorespiratory fitness (MD = −7.59 mL·kg·min, Z = 4.94, *p* < 0.001] [71,74,75,83,84], with substantial heterogeneity (I^2^ = 96%), but no indication of publication bias (*p* = 0.623) (Figure S2C: Supplementary File S5). After subgroup analysis, heterogeneity remained at a considerable level (I^2^ = 72%) for non-treadmill testing. Not meeting the minimum cardiorespiratory fitness level had a significant positive pooled random effect on age in firefighters (MD = 9.33 years, Z = 5.94, *p* < 0.001) [30,40,69] (Figure 2B), with considerable heterogeneity present (I^2^ = 65%) between studies; however, there was no evidence of publication bias (Egger’s test *p* = 0.263) (Figure S2D: Supplementary File S5). After subgroup analysis, heterogeneity was reduced to 0% when a bicycle ergometer testing protocol was used.

### 3.3. The Association between Obesity and Cardiorespiratory Fitness in Firefighters

**Figure 3:**
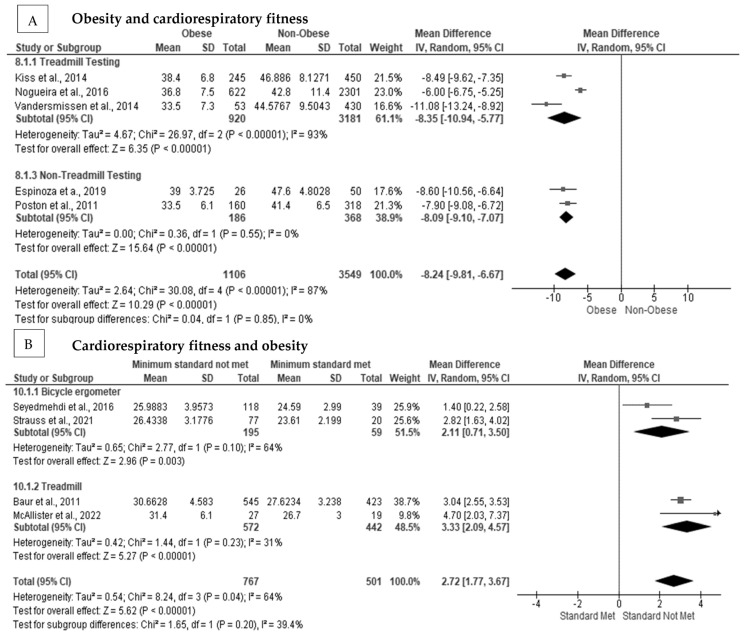
The association between obesity status on cardiorespiratory fitness in firefighters: (**A**) The association between obesity status and cardiorespiratory fitness with subgroup analysis [19,73,75,83,85]; (**B**) the association between cardiorespiratory fitness and BMI with subgroup analysis [30,40,69,79].

Obese firefighters had reduced cardiorespiratory fitness, with many of them not meeting the minimum recommended requirement of 42 mL·kg·min for active duty [19,49,72,73,75,79,83,84,85,86]. In Figure 3A, we show that obesity had a significant negative pooled random effect on cardiorespiratory fitness (MD = −8.24 mL·kg·min, Z = 10.29, *p* < 0.001) [19,73,75,83,85], with considerable heterogeneity between studies (I^2^ = 87%), and no indication of publication bias (Egger’s Test *p* = 0.089) (Figure S3C: Supplementary File S5). After subgroup analysis, there was homogeneity (I^2^ = 0%) present between studies when cardiorespiratory fitness was assessed using a treadmill protocol. The effect remained for obese as compared with non-obese firefighters on cardiorespiratory fitness (MD = −8.09 mL·kg·min, Z = 15.64, *p* < 0.001) [73,85]. In Figure 3B, not meeting the minimum cardiorespiratory fitness level had a significant positive pooled random effect on BMI (MD = 2.72 kg·m^−2^, Z = 5.62, *p* < 0.001) [30,40,69,79], with moderate heterogeneity between studies (I^2^ = 64%); however, no indication of publication bias was seen (Egger’s test *p* = 0.598) (Figure S3D: Supplementary File S5). After subgroup analysis, heterogeneity was reduced (I^2^ = 31%) when a treadmill testing protocol was used to estimate cardiorespiratory fitness.

### 3.4. The Effect of Cardiorespiratory Fitness on Blood Pressure in Firefighters

**Figure 4:**
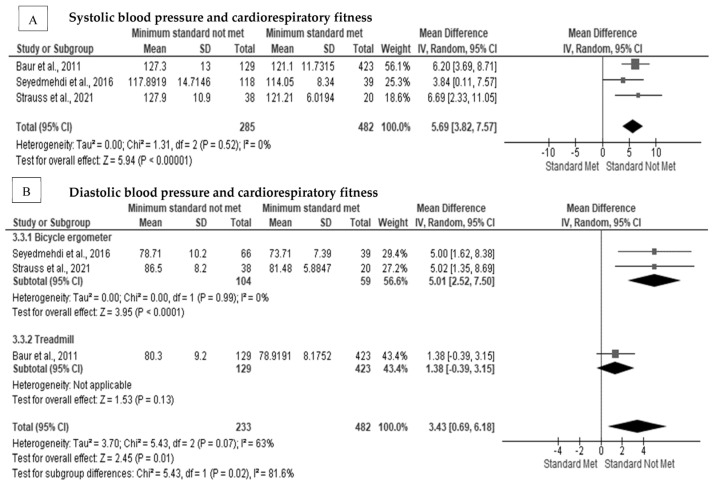
The association between cardiorespiratory fitness and blood pressure in firefighters: (**A**) The association between cardiorespiratory fitness and systolic blood pressure [30,40,69]; (**B**) the association between cardiorespiratory fitness and diastolic blood pressure with subgroup analysis [30,40,69].

Systolic and diastolic blood pressure significantly and inversely influenced the cardiorespiratory fitness in firefighters [30,40,69,72,73,79]. Furthermore, higher blood pressure measurements reduced the overall cardiorespiratory fitness in firefighters since those with higher blood pressure were less likely to meet the minimum cardiorespiratory requirements for firefighting. In Figure 4A, not meeting the minimum cardiorespiratory fitness level had a significant positive pooled random effect on systolic blood pressure (MD = 5.69 mm Hg, Z = 5.94, *p* < 0.001) [30,40,69], with no heterogeneity between studies (I^2^ = 0%) (Figure S4C, Supplementary File S5). Not meeting the minimum cardiorespiratory fitness level had a significant positive pooled random effect on diastolic blood pressure in firefighters (MD = 3.43 mm Hg, Z = 2.45, *p* = 0.01) (Figure 4B) [30,40,69] with considerable heterogeneity present (I^2^ = 63%) between studies; however, there was no evidence of publication bias (Egger’s test *p* = 0.269) (Figure S4D, Supplementary File S5). After subgroup analysis, heterogeneity was reduced to 0% when a bicycle ergometer testing protocol was used. The effect remained significant for cardiorespiratory fitness levels on diastolic blood pressure after subgroup analysis (MD = 5.01 mm Hg, Z = 3.95, *p* < 0.001) [30,40].

### 3.5. The Effect of Cardiorespiratory Fitness on Blood Cholesterol Concentration in Firefighters

**Figure 5:**
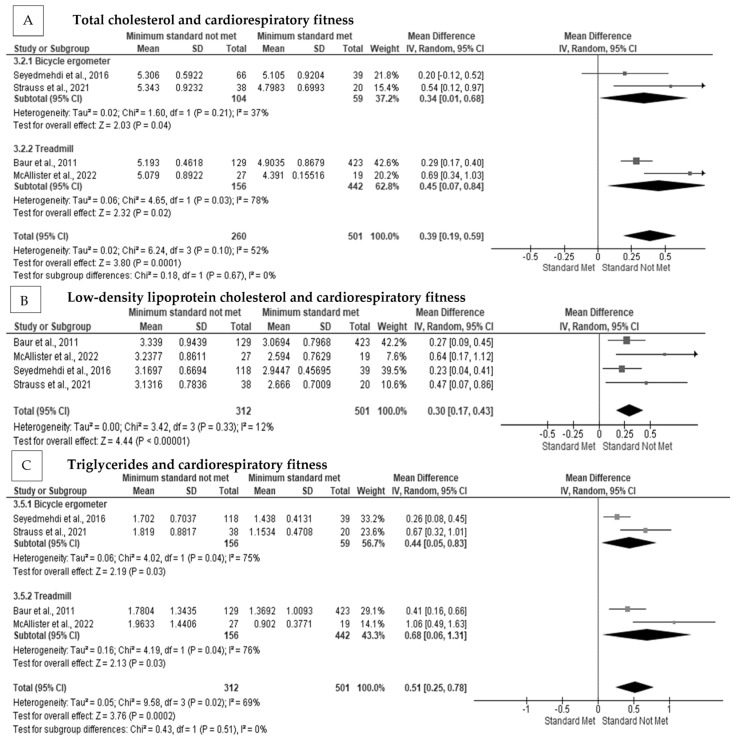
The association between cardiorespiratory fitness and blood lipid concentrations in firefighters: (**A**) The association between cardiorespiratory fitness and total cholesterol concentration with subgroup analysis [30,40,69,79]; (**B**) the association between cardiorespiratory fitness and low-density lipoprotein concentration with subgroup analysis [30,40,69,79]; (**C**) the association between cardiorespiratory fitness and triglyceride concentration with subgroup analysis [30,40,69,79].

Total cholesterol was significantly related to cardiorespiratory fitness in firefighters [30,40,69,79]. In addition, LDL-C [30,40,69,79] and triglycerides [30,40,69,79] were significantly and negatively related to cardiorespiratory fitness, with those firefighters being less likely to meet the minimum cardiorespiratory fitness requirements for firefighting. The meta-analysis indicated that there was a significant positive pooled random effect for not meeting the minimum cardiorespiratory fitness level on total cholesterol concentration in firefighters (MD = 0.39 mmol·L, Z = 3.80, *p* < 0.001) [30,40,69,79] (Figure 5A), with moderate heterogeneity among the studies (I^2^ = 52%), but there was no indication of publication bias (Egger’s Test *p* = 0.312, Figure S5D, Supplementary File S5). After subgroup analysis, heterogeneity was reduced (I^2^ = 37%) when testing used a bicycle ergometer protocol but remained moderate. Not meeting the minimum cardiorespiratory fitness level had a significant pooled random effect on LDL-C concentration (MD = 0.30 mmol·L, Z = 4.44, *p* < 0.001) [30,40,69,79] (Figure 5B), with low heterogeneity among the included studies (I^2^ = 12%). There was a significant pooled effect for not meeting the minimum cardiorespiratory fitness on triglyceride concentration in firefighters (MD = 0.51 mmol·L, Z = 3.76, *p* < 0.001] [30,40,69,79] (Figure 5C), with considerable heterogeneity (I^2^ = 69%) among the studies; however, there was no evidence of publication bias (Egger’s test *p* = 0.144) (Figure S5F, Supplementary File S5). Heterogeneity was not removed following subgroup analysis (I^2^ = 75%).

### 3.6. The Effect of Cardiorespiratory Fitness on Blood Glucose in Firefighters

**Figure 6:**
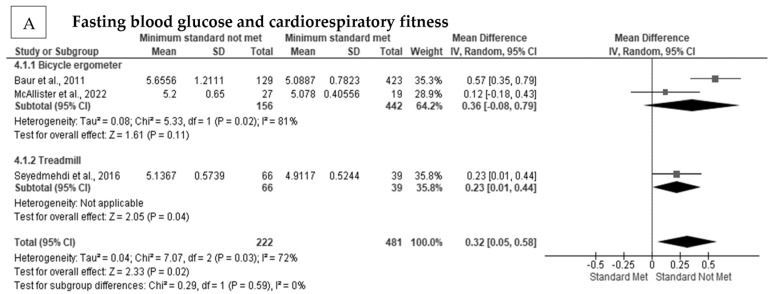
The association between cardiorespiratory fitness and blood glucose in firefighters: (**A**) The association between cardiorespiratory fitness and blood glucose concentration [30,69,79].

Blood glucose was significantly and negatively related to cardiorespiratory fitness in firefighters [30,69,73,79]. There was a significant positive pooled random effect for cardiorespiratory fitness on blood glucose concentration in firefighters (MD = 0.32 mmol·L, Z = 2.33, *p* < 0.001) [30,69,79] (Figure 6A). There was considerable heterogeneity (I^2^ = 72%) present among the studies (Figure S6B, Supplementary File S5), but there was no indication of publication bias (Egger’s test *p* = 0.362). After subgroup analysis, heterogeneity was not reduced (I^2^ = 81%).

### 3.7. The Associations between Cigarette Smoking, Physical Inactivity, CVD Risk, and Heart Rate Variability and Cardiorespiratory Fitness in Firefighters

Punakallio et al. [82] and Durand et al. [35] reported that cigarette smoking was significantly associated with cardiorespiratory fitness in firefighters. Physical inactivity was significantly related to cardiorespiratory fitness in firefighters [35]. Li et al. [77] reported that overall, the 10-year ASCVD risk was not significantly related to cardiorespiratory fitness, after controlling for BF% in firefighters. Metabolic syndrome and an increasing number of cardiometabolic risk factors were significantly and negatively related to cardiorespiratory fitness in firefighters [41,70,78]. Porto et al. [80] reported that the number of times the change in successive normal sinus intervals exceeds 50 ms (PNN50), the root mean square of successive differences between normal heartbeats (rMSSD), and low-frequency and high-frequency ratio (LHR) were significantly different between cardiorespiratory fitness levels.

### 3.8. Correlations between Cardiovascular Disease Risk Factors and Cardiorespiratory Fitness

In Table 2, there was a significantly low negative correlation between age and cardiorespiratory fitness (R = −0.471, *p* < 0.001) [72,73,75], with moderate heterogeneity (I^2^ = 47%). After subgroup analysis, homogeneity (I^2^ = 0%) was present among the studies, when treadmill testing was used to assess cardiorespiratory fitness. There was a significantly moderate negative correlation between obesity and cardiorespiratory fitness (R = −0.595, *p* < 0.001) [49,72,73,75,76,84,86] with substantial heterogeneity (I^2^ = 77%) among the studies. In subgroup analysis, there was no heterogeneity between studies using a cycle ergometer for testing (I^2^ = 0%). There was a significantly high negative correlation between central obesity and cardiorespiratory fitness (R = −0.715, *p* < 0.001) [49,73], with considerable heterogeneity (I^2^ = 80%) among the studies. There was a significantly moderate negative correlation between body-fat percentage and cardiorespiratory fitness (R = −0.663, *p* < 0.001) [72,73,76,84], moderate heterogeneity (I^2^ = 55%) among the studies, and no indication of publication bias (Egger’s test *p* = 0.455). Heterogeneity was reduced (I^2^ = 27%) following subgroup analysis on testing procedures that used a cycle ergometer. There was a significantly negligible negative correlation between systolic blood pressure and cardiorespiratory fitness (R = −0.190, *p* = 0.007) [72,73,84], with homogeneity (I^2^ = 0%) present among the studies. There was a significantly low negative correlation between diastolic blood pressure and cardiorespiratory fitness (R = −0.267, *p* = 0.028) [72,73,84], with moderate heterogeneity (I^2^ = 47%) present among the studies and no indication of publication bias (Egger’s test *p* = 0.089). Heterogeneity was not reduced in subgroup analysis.

## 4. Discussion

### 4.1. Summary of Evidence

To the best of the authors’ knowledge, this is the first systematic review and meta-analysis performed to examine the relationship between CVD risk factors and cardiorespiratory fitness in firefighters. The results indicated that CVD risk factors had a statistically significant and inverse association with cardiorespiratory fitness in firefighters. This was particularly true for age, obesity, blood pressure, and blood lipid concentration. Collectively, having a better overall cardiovascular disease risk profile may have a compounded effect on firefighters’ health, significantly improving their overall cardiorespiratory fitness and reducing their on-duty risks.

### 4.2. Cardiovascular Disease Risk Factors and Cardiorespiratory Fitness

Aged firefighters had poorer cardiorespiratory fitness as compared with younger firefighters. In addition, firefighters that did not meet the minimum required cardiorespiratory fitness were older than those who met the minimum required cardiorespiratory fitness level. Aging causes a decrease in vascular elasticity, which may negatively affect blood flow toward muscles [87,88]. In addition, the atrial and ventricular chambers of the heart show reduced elasticity [46,89], which negatively affects stroke volume and reduces blood flow toward the working muscles [88,89]. Furthermore, aging has been shown to reduce muscle functions, particularly those related to muscular force production [46]. Research has indicated that firefighters tend to become more physically inactive as they age, which may assist in the steady decline in cardiorespiratory fitness seen in this population with time [35,48,49].

The results of our systematic review and meta-analysis indicated that obese firefighters had a significantly lower cardiorespiratory fitness level as compared with non-obese firefighters. In addition, firefighters that did not meet the minimum cardiorespiratory fitness levels had a higher BMI than those that met the requirement. Previous systematic reviews have indicated that obesity significantly reduced cardiorespiratory fitness in sportsmen [90,91] and emergency occupations alike [8,92,93]. Moreover, obesity has been shown to increase the incidence of duty-related fatalities in firefighters [2]. Obesity increases non-functional mass in firefighters that are required to be carried. Increased peripheral resistance subsequently increases blood pressure, which reduces atrial preload, negatively affecting stroke volume and oxygen uptake to working muscles [47,94]. Increased fat mass increases the effort of respiratory muscles to expand the rib cage, reducing the available oxygen content toward working muscles [95]. Firefighters are required to carry additional weight while on duty [5], and therefore, firefighters are required to have a much higher cardiorespiratory fitness to cope with these stressors [6,83,96]. Obese firefighters who carry additional heavy protective gear may be significantly predisposed to poorer occupational performance [6,83,96] and significantly higher cardiorespiratory strain [3].

Firefighters that met the minimum cardiorespiratory standard had lower systolic and diastolic blood pressure than firefighters that did not meet the minimum requirements. Increased blood pressure, due to reduced vascular elasticity and increased total peripheral resistance, directly reduced stroke volume [97]. As mentioned previously, a reduction in stroke volume diminishes the quantity of oxygen transported to working muscles, limiting oxygen usage and energy production [43,97]. Firefighters should maintain normal and preferably optimal blood pressure levels throughout their careers to maintain adequate cardiorespiratory fitness levels, through multiple interventions.

In the present study, firefighters that did not meet the minimum cardiorespiratory fitness standard for firefighting had significantly higher concentrations for total cholesterol, LDL-C, triglycerides, and blood glucose. The current results were supported by previous literature that indicated physical fitness was significantly related to LDL-C and triglyceride concentrations [45,98,99]. Rumora et al. [100] reported that high LDL-C levels impaired mitochondrial function, by reducing oxygen uptake and subsequently reducing aerobic capacity. Physical activity was reported to improve blood lipid concentrations, specifically in reducing LDL-C and increasing HDL-C [99]. In addition, previous reviews have indicated that blood glucose and diabetes were significantly related to cardiorespiratory fitness [44,101]. The prevalence of diabetes is relatively low in firefighters; however, diabetes may have the highest overall impact on their overall cardiorespiratory fitness levels among the haematological parameters [9,12,27,30,33,69,102].

In previous studies, cigarette smoking and physical activity were investigated in relation to cardiorespiratory fitness in firefighters; however, insufficient studies were available to perform a meta-analysis. The results indicated that cigarette smoking was significantly related to declining cardiorespiratory fitness levels in firefighters over time. This was supported by the results of previous systematic reviews that indicated cigarette smoking negatively affected the cardiorespiratory fitness of healthy adult males [103]. In addition, exposure to passive smoke was also related to reduced cardiorespiratory fitness [103]. Physical inactivity, especially in obese firefighters, was significantly related to poorer cardiorespiratory fitness in firefighters. This is supported by a previous systematic review in young adults, where longer duration of physical activity sessions and higher frequency of physical activity were significantly related to cardiorespiratory fitness [104]. Only one study was found that investigated the overall effect of cardiovascular risk status on cardiorespiratory fitness in firefighters, indicating that a significant gap exists. This has been proven in previous research where higher cardiorespiratory fitness improved overall cardiovascular risk and all-cause mortality [105,106]. Similar to overall cardiovascular disease risk, only one study was found that investigated heart rate variability and cardiorespiratory fitness. The results indicated that heart rate variability increased as cardiorespiratory fitness increased, suggesting that optimal cardiovascular health was related to higher levels of cardiorespiratory fitness [80].

### 4.3. Strengths and Limitations

The overall quality of the studies that were included in our review was high, with most studies being above a score of eighteen. This study provided valuable evidence in an understudied research field in firefighters. An inherent weakness is that insufficient studies have investigated all the cardiovascular disease risk factors in firefighters, specifically cigarette smoking, physical inactivity, and a family history of cardiovascular disease. In addition, only one study used an overall risk score to determine cardiovascular risk on cardiorespiratory fitness. Several studies that were included did not include a selection procedure that would allow researchers to choose individuals who were representative of the intended population and the sample frame was not taken from an appropriate population base. A limited number of studies were available for many of the analyses, reducing the strength of the inferences that could be made. Lastly, cardiorespiratory fitness was not consistently measured in the same way, which likely contributed to the heterogeneity of results that could not be explained through subgroup analysis.

## 5. Conclusions

The current systematic review and meta-analysis support previous findings suggesting that firefighters need to maintain optimum cardiovascular health and cardiorespiratory fitness throughout their careers. As the reciprocal relationship indicated, this will reduce the likelihood of firefighters’ cardiorespiratory fitness dropping below the minimum requirements for firefighting. Decreased cardiorespiratory fitness was attributed to the development and progression of CVD risk factors, which was most notable in firefighters not meeting the minimum recommended fitness level. Given that firefighters actively maintain their cardiorespiratory fitness levels, this is expected to have a positive effect on their cardiovascular health, subsequently reducing the risk of CVD-related morbidity and mortality. Fire and Rescue departments should promote regular physical activity and behavioral medication programs designed to not only increase the cardiorespiratory fitness of firefighters but improve their overall cardiovascular health status. It is recommended that fire departments should adopt scheduled physical activity or exercise programs while firefighters are on-duty and should ensure that firefighters are regularly assessed to measure their cardiorespiratory health. Future research should focus on the collective effect that an increased CVD risk status may have on cardiorespiratory fitness in firefighters. In addition, more studies should be conducted on cardiorespiratory fitness in relation to total fitness. i.e., muscular endurance, muscular strength, flexibility, and body composition. Few studies investigated the relationship between heart rate variability and cardiorespiratory fitness, indicating a significant research gap in this particular area.

## 6. Patents

### Protocol Registration

Details of the protocol for this systematic review were registered on PROSPERO (CRD42022330510) and can be accessed at: https://www.crd.york.ac.uk/prospero/display_record.php?ID=CRD42022330510 (accessed on 18 December 2022).

## Figures and Tables

**Table 1:** Characteristics of included studies in the narrative synthesis and quantitative meta-analysis.

References	Study Design and Setting	Sample	Cardiovascular Disease Risk Factors	Cardiorespiratory Fitness	Performance Measures	Outcome
Atikah et al. [67]	Cross-sectional study Malaysia, Kebangsaan	385 Male firefighters	Age: N/A BMI: N/A	VO_2_max: 26.49 ± 5.14 mL·kg·min	20 m shuttle run test	The cardiorespiratory fitness of firefighters aged between 20 and 28 years old was significantly higher as compared with firefighters aged between 29 and 37 years old and between 38 and 46 years old.
Baur et al. [68]	Cross-sectional study USA, Massachusetts	957 Male firefighters	Age: 39.6 ± 8.5 years BMI: 29.4 ± 4.3 BF%: 21.6 ± 6.0	MaxMETs: 12.0 ± 1.9 METs	Maximal treadmill exercise stress test	The number of metabolic syndrome risk factors was significantly different between maximum MET groups (<0.00001). Metabolic syndrome was a significant predictor of cardiorespiratory fitness (CRF).
Baur et al. [69]	Cross-sectional study USA, Massachusetts	968 Male firefighters	Age: 39.5 ± 8.6 years BMI: 29.3 ± 4.3 kg·m^−2^ BF%: 21.0 ± 5.6% Cigarette smokers: 23.9% SBP: 122.6 ± 12.0 mmHg DBP: 78.6 ± 8.6 mmHg TC: 5.02 ± 0.96 mmol·L LDL-C: 3.22 ± 0.83 mmol·L HDL-C: 1.13 ± 0.30 mmol·L TG: 1.5 ± 1.3 mmol·L	METs: 12.0 ± 1.9 METs	Maximal treadmill exercise stress test	There were significant associations between max METs and age, BMI, systolic blood pressure (SBP), diastolic blood pressure (DBP), triglycerides, total cholesterol, low-density lipoprotein cholesterol (LDL-C), and blood glucose.
Baur et al. [70]	Cross-sectional study USA	804 Male firefighters	Age: 37.4 ± 8.4 years BMI: 29.3 ± 4.4 kg·m^−2^	MaxMETs: 10.7 ± 2.0	Maximal treadmill stress test	Cardiorespiratory was significantly related to age, physical activity, and BMI in firefighters.
Barry et al. [49]	Cross-sectional study USA	30 Male firefighters	Age: 34.45 ± 7.15 years Height: 180.74 ± 6.80 cm Body mass: 94.70 ± 10.65 kgs BMI: 28.97 ± 2.52 kg·m^−2^ WC: 96.48 ± 7.95 cm	VO_2_max: 40.82 ± 6.95 mL·kg·min	Treadmill graded exercise stress test	There were significant correlations between sedentary time (r = −0.62, *p* < 0.001), vigorous physical activity time (r = −0.48, *p* < 0.001), waist circumference (WC) (r = −0.55, *p* < 0.01), and BMI (r = −0.53, *p* < 0.01) and VO_2_max.
Cameron et al. [71]	Cross-sectional study USA, San Diego	1169 Male firefighters	Age: 38.1 ± 0.3 years Height: 178.79 ± 6.65 cm Weight: 88.42 ± 13.36 kgs BMI: 27.65 ± 3.79 kg·m^−2^	METs: 13.96 ± 2.43 METs	Maximal treadmill graded exercise stress test	Cardiorespiratory fitness was significantly different between age groups in firefighters (*p* < 0.001).
Delisle et al. [72]	Cross-sectional study USA, Florida	30 Male and female firefighters	Age: 31.9 ± 6.4 years BF%: 26.0 ± 6.4% BMI: 27.2 ± 3.8 kg·m^−2^	VO_2_max: 44.6 ± 3.9 mL·kg·min	Bruce treadmill protocol	Cardiorespiratory fitness was significantly and moderately associated with percentage body fat (r = −0.7353, *p* = 0.0001), DBP (r = −0.541, *p* = 0.0035), BMI (r = −0.5445, *p* = 0.003), 1-min recovery HR (r = 0.537, *p* = 0.0038), and body composition (r = −0.5178; *p* = 0.008).
Donovan et al. [41]	Cross-sectional study USA, Colorado	214 Male firefighters	Age: 39 ± 9 Height: 179 ± 6 cm Weight: 88 ± 15 kg BMI: 28 ± 4 kg·m^−2^ BF%: 21 ± 7% WC: 94 ± 11 cm SBP: 129 ± 12 mmHg DBP: 83 ± 8 mmHg TG: 1.4 ± 0.9 mmol·L HDL-C: 1.2 ± 0.3 mmol·L FGL: 4.8 ± 0.5 mmol·L CS: 3.7%	VO_2_max: 46.3 ± 6.1 mL·kg·min	Bruce treadmill protocol	After controlling for age, cardiorespiratory fitness was inversely associated with increasing metabolic abnormalities (*p* < 0.001). Estimated VO_2_max values for firefighters with 0, 1, 2, and 3 metabolic abnormalities were 47.8 mL·kg·min, 47.7 mL·kg·min, 45.2 mL·kg·min, and 43.6 mL·kg·min, respectively. The estimated VO_2max_ for subjects with two, three, or more metabolic abnormalities were found to be significantly lower than that of subjects with zero or one metabolic abnormality.
Durand et al. [35]	Cross-sectional cohort study USA, Kansas and Missouri	527 Full-time male firefighters	Age: 37.2 ± 8.6 years BMI: 29.3 ± 4.5 kg·m^−2^	METs: 12.7 ± 1.6 METs	Bruce or modified Bruce protocols	Among the four CRF outcome variables, max-METs was significantly different among the three PA categories after adjusting for age, BMI, and smoking status (*p* < 0.001). The association with CRF was strong across all three measures of PA dimensions, as well as with total weekly aerobic exercise.
Espinoza et al. [73]	Cross-sectional study Chile., South America	76 Volunteer male firefighters	Age: 27.5 years Height: 172 cm BMI: 27.7 kg·m^−2^ SBP: 120 mmHg DBP: 73 mmHg FGL: 5.4 mmol·L	VO_2_max: 44 mL·kg·min	Leger test	CRF was negatively correlated with age, BMI, WC, BF%, SBP, DBP, and blood glucose. CRF was significantly different among normal-weight and obese firefighters.
Kirlin et al. [74]	Cross-sectional study USA, San Diego	96 Full-time female firefighters	Age group: 25–34 vs. 35–44 vs. 45–54 vs. 55+ years Height: 170.7 ± 4.9 vs. 169.4 ± 5.4 168.4 ± 5.7 vs. 167.9 ± 4.3 cm Weight: 72.2 ± 10.2 vs. 74.2 ± 13.4 vs. 73.6 ± 13.4 vs. 68.7 ± 9.3 kgs BMI: 24.7 ± 3.3 vs. 25.8 ± 4.4 vs. 25.9 ± 4.4 vs. 24.4 ± 3.8 kg·m^−2^ BF%: 22.9 ± 7.0 vs. 25.1 ± 8.2 vs. 26.7 ± 7.7 vs. 24.0 ± 6.4%	VO_2_max: 50.9 ± 7.4 vs. 45.0 ± 7.1 vs. 42.8 ± 6.4 vs. 45.2 ± 5.1 METs: 14.6 ± 2.1 vs. 12.9 ± 2.0 vs. 12.2 ± 1.8 vs. 12.9 ± 1.5 METs	Graded exercise test	CRF decreased significantly across the age groups. Post hoc analysis showed a significantly lower relative VO_2_max in the 35–44 age group as compared with the 25–34 age group and in the 45–54 age group as compared with the 25–34 age group. Post hoc analysis of absolute VO_2_max revealed a significantly higher CRF in the 25–34 age group as compared with the 35–44 group, the 45–54 age group, and the 55+ group.
Kiss et al. [75]	Cross-sectional study Belgium, Ghent	1249 Firefighters	Age: 38 ± 10 years BF%: 24.6 ± 7.0% BMI: 26.0 ± 3.8 kg·m^−2^	VO_2_max: 46.5 ± 8.8 mL·kg·min	Maximal treadmill exercise stress test	Cardiorespiratory fitness was significantly different between age groups, BF% categories, and BMI categories in firefighters. In addition, age, BF%, and BMI were significant predictors of cardiorespiratory fitness.
Houck et al. [76]	Cross-sectional study USA, New Mexico	80 Male and female firefighters	Age: 34.9 ± 7.9 years Height: 178.2 ± 6.2 cm Weight: 85.0 ± 12.0 kgs BMI: 26.7 ± 3.0 kg·m^−2^ BF%: 18.7 ± 6.3% SBP: 122.0 ± 8.4 mmHg DBP: 78.3 ± 7.2 mmHg	VO_2_max: 38.4 ± 6.8 mL·kg·min	Graded exercise test Bicycle ergometer test	Cardiorespiratory fitness was significantly and negatively associated with BF% (r = −0.597), BMI (r = −0.497), maximal SBP (r = −0.305), maximal DBP (r = 0.262), and resting HR (r = 0.320). Lean body mass was significantly positively correlated with cardiorespiratory fitness (r = 0.576).
Li et al. [77]	Cross-sectional study USA, Colorado	294 Full-time male and female firefighters	Age: 46.88 ± 5.67 years Height: 1.78 ± 0.10 m Weight: 89.2 ± 17.3 kgs BMI: 28.6 ± 10.1 kg·m^−2^ BF%: 23.8 ± 7.01%	VO_2_ max: 44.5 ± 5.94 mL·kg·min	Maximal exercise test	Results of bivariate logistic regression show that %BF (odds ratio [OR] = 1.24, *p* < 0.01), estimated VO_2_ max (OR = 0.90, *p* < 0.05), and metabolic syndrome (OR = 2.66, *p* < 0.05). The age group (*p* < 0.001) was significantly related to 10-year ASCVD risk. BMI and sex were not significantly associated with 10-year ASCVD risk. No significant association was found between VO_2_ max and 10-year ASCVD risk.
Li et al. [78]	Cross-sectional study USA, Colorado	1099 Male and female firefighters	Age: 37.2 ± 9.8 years Male: 37.1 ± 9.8; female 38.0 ± 10.1 BF%: female: 21.1 ± 7.9%; male: 18.4 ± 6.7%	VO_2_max: 46.9 ± 6.8 mL·kg·min	Bruce protocol	In total, 49% of firefighters did not meet the minimum cardiorespiratory fitness level of 42.0 mL·kg·min. VO_2_ max, body fat values, and age group were significantly associated with the number of metabolic syndrome components among males and body fat values, but VO_2_max and age group, were not significantly associated with the number of metabolic syndrome components among females. VO_2_ max (*p* < 0.001) was negatively associated with the number of metabolic syndrome components.
Seyedmedi et al. [30]	Cross-sectional study Iran, Tehran	157 Male firefighters	Age: 34.18 years BMI: 25.61 kg·m^−2^ Aerobic fitness: 33.76 mL·kg^−1^·min^−1^ SBP: 116.93 mmHg DBP: 76.03 mmHg TC: 5.22 ± 0.72 mmol·L LDL-C: 3.11 ± 0.63 mmol·L HDL-C: 1.02 ± 0.17 mmol·L TG: 1.6 ± 0.7 mmol·L FGL: 5.0 ± 0.6 mmol·L	METs: 9.64 METs	YMCA bicycle ergometer test	Significant differences between individuals with >11 MET versus individuals with <9 MET for all factors with the exception of total cholesterol, fasting blood sugar, and SBP. The high CRF group was significantly younger with lower BMI, triglycerides, LDL, resting heart rate, DBP, and higher HDL. The frequency of subjects with CVD risk factors in the group with AF < 9 MET was significantly higher than that in the group with AF ≥ 9 MET (*p* < 0.05) for all factors except triglycerides. Individuals with low AF were more than 5 times as likely to smoke, not participate in physical activity, and have higher LDL-C levels than firefighters with high AF.
McAllister et al. [79]	Cross-sectional study USA, Texas	46 Full-time firefighters	Age: 37.2 ± 8.9 BF%: 24.1 ± 5.4 BMI: 29.5 ± 5.5 FGL: 5.1 ± 0.6 mmol·L TC: 4.79 ± 0.77 mmol·L LDL-C: 2.97 ± 0.87 mmol·L HDL-C: 1.20 ± 0.34 mmol·L TG: 1.5 ± 1.2 mmol·L	VO_2_max: 35.0 ± 9.6	Bruce protocol	There were significant differences among BMI (*p* < 0.01), BF% (*p* < 0.001), cholesterol (*p* < 0.05), triglycerides (*p* < 0.001), HDL-C (*p* < 0.05), and LDL-C (*p* < 0.01) between the low fit and high fit groups.
Nogueira et al. [19]	Cross-sectional studyBrazil	4237 Full-time male firefighters	Age: 39 (22–49) years BMI: 26.6 (16.9–43.8) kg·m^−2^ WC: 90.0 cm (55.0–136.0) BAI = 24.9 (10.5–38.3) BF% = 21.7% (14.0–34.3%)	VO_2_max: 42.4 mL·kg·min	12 min Cooper test	VO_2_max was negatively correlated with age (r = 20.21, *p* < 0.001), WC (r = 20.50, *p* < 0.001), BMI (r = −0.45, *p* < 0.001), and BAI (r = −0.35, *p* < 0.001). The proportion of obese FF among the less fit firefighters was 5.5-fold higher than among the fittest group. Poor cardiorespiratory fitness (<12 METs) was associated with all indices of obesity, i.e., BMI (*p* < 0.001), BAI (*p* < 0.001), BF% (*p* <0.001), and WC (*p* < 0.001).
Perroni et al. [42]	Cross-sectional study Italy, Rome	161 Male firefighters	Age: 33 ± 7 years Height: 176 ± 6 cm Weight: 75.8 ± 8.4 kgs BMI: 24.4 ± 2.3 kg·m^−2^	VO_2_max: 51.8 ± 6.8 mL·kg·min	Queensland College step test	Age was significantly related to cardiorespiratory fitness in firefighters.
Porto et al. [80]	Cross-sectional study Brazil	38 Firefighters	Age: 41 years BMI: 26.1 kg·m^−2^	VO_2_max: 42.4 mL·kg·min	SRPA questionnaire estimated VO_2_max	PNN50, rMSSD, and LHR were significantly different between cardiorespiratory fitness categories. Fitter firefighters had better heart rate variability.
Poston et al. [81]	Cross-sectional study USA, Missouri	478 Full-time and 199 volunteer firefighters	Age: 38.64 ± 10.57 years Height: 178.45 ± 6.45 cm Weight: 92.33 ± 16.47 kgs BMI: 28.86 ± 4.83 kg·m^−2^ BF%: 25.56 ± 6.95% SBP: 125.9 ± 13.2 mmHg DBP: 79.2 ± 10.6 mmHg TC: 4.06 ± 1.03 mmol·L LDL-C: 2.6 ± 0.9 mmol·L HDL-C: 0.98 ± 0.32 mmol·L TG: 1.4 ± 0.9 mmol·L	METs: 10.9 ± 2.5 VO_2_max: 37.8 ± 8.1 mL·kg·min	Self-report of physical activity questionnaire	Obese firefighters had significantly lower cardiorespiratory fitness than non-obese firefighters.
Punakallio et al. [82]	Longitudinal study Finland, Helsinki	78 Full-time male firefighters	Age group: 30–34 vs. 40–44 years Age: 32.5 ± 1.5 vs. 41.8 ± 1.4 years Height: 179.8 ± 6.0 vs. 176.6 ± 5.5 cm Weight: 83.6 ± 8.0 vs. 83.6 ± 8.4 kgs BMI: 25.9 ± 2.2 vs. 26.9 ± 2.7 kg·m^−2^ Experience: 10.4 ± 2.5 vs. 19.3 ± 2.3 years	VO_2_max: 41.7 ± 6.42 vs. 36.0 ± 5.97 mL·kg·min	Incremental exercise bicycle ergometer test	Age-standardized regular smoking (*p* = 0.048) and the sum of variables related to lifestyle factors (*p* = 0.034) significantly predicted absolute VO_2_max after 13 years.
Strauss et al. [40]	Cross-sectional study Germany, Westphalia	97 Full-time male firefighters	Age: 40.5 ± 9.0 years BMI: 25.9 ± 3.2 kg·m^−2^ BF%: 17.7 ± 6.2% WC: 89.8 ± 10.0 cm Experience: 16.3 ± 9.1 years SBP: 126.4 ± 9.8 mmHg DBP: 84.1 ± 7.4 mmHg TC: 5.1 ± 0.9 mmol·L LDL-C: 2.9 ± 0.8 mmol·L HDL-C: 1.4 ± 0.3 mmol·L TG: 1.6 ± 0.8 mmol·L	METs: 10.7 ± 1.8 METs	Bicycle spiroergometric exercise stress test	Higher lipid concentrations, DBP, SBP, heart rates, WC, BF%, and years of work experience were inversely related to lower cardiorespiratory fitness levels. Significant associations were present between higher cardiorespiratory fitness and lower BMI (*p* < 0.0001), WC (*p* < 0.0001), BF% (*p* < 0.0001), SBP (*p* = 0.0061), triglycerides (*p* = 0.0018), and total cholesterol levels (*p* = 0.0443).
Vandersmissen et al. [83]	Cross-sectional study Belgium	605 Full-time male firefighters	Age: 40.4 ± 11.5 years BMI: 25.9 ± 3.4 kg·m^−2^ WC: 92.3 ± 10.3 cm HRmax: 99.7 ± 7.6 bpm	VO_2_max: 43.3 ± 9.8 mL·kg·min	Maximal treadmill and bicycle ergometer exercise stress test	Cardiorespiratory capacity was significantly related to age (*p* < 0.001), BMI (*p* < 0.001), and WC (*p* < 0.001). Firefighters older than 45 years and those that were obese or had central obesity had a mean VO_2_max under 42 mL·kg·min.
Vicente et al. [84]	Cross-sectional study Italy	104 Full-time male firefighters	Age: 47.1 ± 6.8 years BMI: 26.6 ± 2.5 kg·m^−2^ BF%: 22.9 ± 5.0 SBP: 125.4 ± 21 mmHg DBP: 88.9 ± 21.6 mmHg	VO_2_max: 45.7.3 ± 7.0 mL·kg·min	Shuttle test	CRF was significantly different between the age group categories (*p* < 0.001). There was a significant negative correlation between CRF and age (r = −0.50, *p* < 0.01).

Note: Units of measurements: m—meters; cm—centimeters; kgs—kilograms; VO_2_—oxygen consumption; VO_2max_—maximum oxygen consumption; HR—heart rate; BMI—body mass index; WC—waist circumference; BF%—body fat percentage; BAI—body adiposity index; kg·m^−2^—kilograms per meter squared; mL·kg·min.- milliliters per kilogram per minute; bmp—beats per minute; SBP—systolic blood pressure; DBP—diastolic blood pressure; TC—total cholesterol; LDL-C—low-density lipoprotein cholesterol; HDL-C—high-density lipoprotein cholesterol; TG—triglycerides; FBG—fasting blood glucose; PNN50—successive normal sinus (NN) intervals exceeding 50 ms, rMSSD—root mean square root of successive differences; LHR—low/high-frequency ratio; ASCVD—atherosclerotic cardiovascular disease.

**Table 2:** The correlations between age, obesity, and blood pressure and cardiorespiratory fitness.

Outcome	No. of Studies	No. of Participants	R (95% CI)	Z Score	*p* (Overall Effect)	Heterogeneity I^2^; Cohen’s Q; *p*-Value	Egger’s Test Intercept (95%CI); *p*	Begg’s Test (τ; *p*)
Age	4	1434	−0.471 (−0.562 to −0.369)	−8.073	<0.001 **	47%; 5.66; 0.129	1.72 (−0.65 to 4.10); 0.089	0.67; 0.174
*Treadmill testing*	2	105	−0.334 (−0.497 to −0.150)	−3.460	0.001 **	0%; 0.24; 0.628	1.40 (--); <0.001	1.00; 0.317
*Gas analysis*	2	1254	−0.451 (−0.654 to −0.188)	−3.215	0.001 **	63%; 2.67; 0.102	1.93 (--); <0.001	1.00; 0.317
Obesity	7	1632	−0.595 (−0.681 to −0.493)	−9.263	<0.001 **	73%; 22.08; <0.001	−1.28 (−4.12 to 1.57); 0.301	−0.19; 0.538
*Treadmill testing*	3	1330	−0.645 (−0.819 to −0.362)	−3.879	<0.001 **	90%; 20.89; <0.001	−2.71 (−42.69 to 37.28); 0.548	−0.33; 0.602
*Non-Treadmill testing*	4	302	−0.560 (−0.634 to −0.476)	−10.769	<0.001 **	0%; 1.03; 0.793	0.59 (−6.08 to 7.27); 0.739	0.33, 0.602
*Gas analysis*	3	245	−0.658 (−0.808 to −0.428)	−4.652	<0.001 **	85%; 13.57; 0.001	−34.02 (−691.86 to 623.82); 0.629	−0.33; 0.602
Central obesity	2	105	−0.715 (−0.884 to −0.3810	−3.543	<0.001 **	80%; 4.96; 0.026	6.43 (--); <0.001	1.00; 0.317
Body-fat percentage	4	290	−0.663 (−0.753 to −0.550)	−8.640	<0.001 **	55%; 6.62; 0.085	−3.26 (−18.49 to 11.98); 0.455	−0.67; 0.174
*Cycle ergometer testing*	2	110	−0.639 (−0.739 to −0.511)	−7.715	<0.001 **	27%; 1.37; 0.241	−3.34 (--); <0.001	−1.00; 0.317
Systolic blood pressure	3	209	−0.190 (−0.319 to −0.053)	−2.716	0.007 **	0%; 0.37; 0.829	−0.49 (−17.06 to 16.08); 0.772	−0.33; 0.602
*Treadmill testing*	2	105	−0.230 (−0.406 to −0.0367)	−2.326	0.020 *	0%; 0.03; 0.854	0.53 (--); <0.001	1.00; 0.317
Diastolic blood pressure	3	209	−0.267 (−0.475 to −0.030)	−2.202	0.028 *	47; 1.72; 0.129	1.72 (−0.65 to 4.10); 0.089	0.67; 0.174
*Treadmill testing*	2	105	−0.375 (−0.622 to −0.059)	−2.310	0.021 *	57%; 2.33; 0.127	−4.41 (--); <0.001	−1.00; 0.317

Note: * indicates significant <0.05; ** indicates significant <0.01. Italics—indicates subgroup analysis.

## Data Availability

All data generated or analyzed during this study are included in the published review article.

## Supplementary Materials

The following supporting information can be downloaded at: https://www.mdpi.com/article/10.3390/ijerph20042816/s1, Supplementary File S1: Search strategry; Supplementary File S2: Data extraction form; Supplementary File S3: Data extraction form; Supplementary File S4: Table S1. critical appraisal of cross-sectional studies (adapted from the appraisal tool for cross-sectional studies checklist); Table S2: Critical Appraisal Skills Programme of cohort and case-controlled studies (adapted from the Critical Appraisal Skills Programme). Supplementary File S5: Figure S2C: Funnel plot for publication bias; Figure S2D: Funnel plot for publication bias; Figure S3C: Forest plot for publication bias; Figure S3D: Forest plot for publication bias; Figure S4C: Forest plot for publication bias; Figure S4D: Forest plot for publication bias; Figure S5D: Forest plot for publication bias; Figure S5E: Forest plot for publication bias; Figure S5F: Forest plot for publication bias; Figure S6B: Forest plot for publication bias.
