# Supplementary material for: Association between Cardiovascular Disease Risk Factors and Cardiorespiratory Fitness in Firefighters: A Systematic Review and Meta-Analysis
Source: Int J Environ Res Public Health. 2023 Feb 5;20(4):2816. doi: 10.3390/ijerph20042816 (PMC9957465; doi:10.3390/ijerph20042816)
Supplement: Supplementary file 1 [file ijerph-20-02816-s001.zip › 1. Supplementary File S1.pdf]

*File S1*

## Search syntax in different databases

### Scopus

#1 ( TITLE-ABS-KEY “firefighter*” ) + OR AND TITLE-ABS-KEY ( "fire and rescue" OR firefighters OR fire fighter OR firefight OR firemen)

#2 AND "cardiovascular" OR “cardiovascular abnormalities” OR “HRV*” OR “heart rate variability*” OR "heart rate interval" OR “aging” OR “RR variability” OR “cycle length variability” OR “heart period variability” OR “autonomic function” OR “vagal control” OR “lipid profile” OR “cholesterol” OR “diabetes” AND “mellitus” OR “blood glucose” OR “age” OR “obesity” AND ( EXCLUDE ( DOCTYPE , "no" ) OR EXCLUDE ( DOCTYPE , "cp" ) OR EXCLUDE ( DOCTYPE , "ch" ) OR EXCLUDE ( DOCTYPE , "bk" ) )

#3 AND “physical fitness” OR “exercise” OR “physical exertion” OR “muscular strength” OR “muscular endurance” OR “aerobic fitness*” OR “cardiorespiratory fitness*” OR “cardiorespiratory capacity*” AND (EXCLUDE ( DOCTYPE , "no" ) OR EXCLUDE ( DOCTYPE , "cp" ) OR EXCLUDE ( DOCTYPE , "ch" ) OR EXCLUDE ( DOCTYPE , "bk" ) )

#4 (#1 AND #2) OR (#1 AND #3) OR (#1 AND #2 AND #3)

### Web of sciences

#1 TOPIC:(Firefighter*)/ ("fire and rescue" OR firefighters OR fire fighter OR firefight OR firemen)

#2 AND TOPIC:("cardiovascular" OR “cardiovascular abnormalities” OR “HRV*” OR “heart rate variability*” OR "heart rate interval" OR “aging” OR “RR variability” OR “cycle length variability” OR “heart period variability” OR “autonomic function” OR “vagal control” OR “lipid profile” OR “cholesterol” OR “diabetes” AND “mellitus” OR “blood glucose” OR “age” OR “obesity”)Refined by: [excluding] DOCUMENT TYPES: (PROCEEDINGS PAPER OR BOOK CHAPTER OR NOTE OR MEETING ABSTRACT)

#3 AND TOPIC:("physical fitness" OR “exercise” OR “physical exertion” OR “muscular strength” OR “muscular endurance” OR “aerobic fitness” OR “cardiorespiratory fitness” OR “cardiorespiratory capacity”) Refined by: [excluding] DOCUMENT TYPES: (PROCEEDINGS PAPER OR BOOK CHAPTER OR NOTE OR MEETING ABSTRACT)

#4 (#1 AND #2) OR (#1 AND #3) OR (#1 AND #2 AND #3)

**ScienceDirect**

#1 (firefighter*:ab,ti OR firefighters*:ab,ti OR fire and rescue personnel*:ab,ti OR firefight*:ab,ti OR fire firefighter*:ab,ti OR firemen*:ab,ti)

#2 AND (cardiovascular*:ab,ti OR ‘cardiovascular abnormalities’:ab,ti” OR ‘HRV’:ab,ti OR ‘heart rate variability’:ab,ti OR ‘heart rate interval’:ab,ti OR ‘aging’:ab,ti OR ‘RR variability’:ab,ti OR ‘cycle length variability’:ab,ti OR ‘heart period variability’:ab,ti OR ‘autonomic function’:ab,ti OR ‘vagal control’:ab,ti OR ‘lipid profile’:ab,ti OR ‘cholesterol’:ab,ti OR ‘diabetes’:ab,ti AND ‘mellitus’:ab,ti OR ‘blood glucose’:ab,ti OR ‘age’:ab,ti OR ‘obesity’:ab,ti)

#3 (physical fitness*:ab,ti OR ‘exercise’:ab,ti OR ‘physical exertion’:ab,ti OR ‘muscular strength’:ab,ti OR ‘muscular endurance’:ab,ti OR ‘aerobic fitness’:ab,ti OR ‘cardiorespiratory fitness’:ab,ti OR ‘cardiorespiratory capacity’:ab,ti)

#4 (#1 AND #2) OR (#1 AND #3) OR (#1 AND #2 AND #3)

**EBSCOHost**

#1 Subject Terms:(“Firefighter” OR "fire and rescue" OR “firefighters” OR “fire fighter” OR “firefight” OR “firemen”) Field:(All text)

#2 AND ("cardiovascular” OR “cardiovascular abnormalities” OR “HRV*” OR “heart rate variability*” OR "heart rate interval" OR “aging” OR “RR variability” OR “cycle length variability” OR “heart period variability” OR “autonomic function” OR “vagal control” OR “lipid profile” OR “cholesterol” OR “diabetes” AND “mellitus” OR “blood glucose” OR “age” OR “obesity”) Field:(All text)

#3 AND (“physical fitness” OR “exercise” OR “physical exertion” OR “muscular strength” OR “muscular endurance” OR “aerobic fitness*” OR “cardiorespiratory fitness*” OR “cardiorespiratory capacity*”) Field:(All text)

#4 (#1 AND #2) OR (#1 AND #3) OR (#1 AND #2 AND #3)
