# Supplementary material for: Association between Cardiovascular Disease Risk Factors and Cardiorespiratory Fitness in Firefighters: A Systematic Review and Meta-Analysis
Source: Int J Environ Res Public Health. 2023 Feb 5;20(4):2816. doi: 10.3390/ijerph20042816 (PMC9957465; doi:10.3390/ijerph20042816)
Supplement: Supplementary file 1 [file ijerph-20-02816-s001.zip › 2. Supplementary File S2_eligbility screening.pdf]

File S2

**General information**	
Title	
Author	
Study type	
Exposure assessed	
Outcome relevance	
Notes:	

**Study eligibility**			
Study characteristics	Eligibility criteria met		
	Yes	No	Unclear
Type of Study			
Type of participants			
Type of exposure			
Type of comparison			
Types of outcome measures			

INCLUDE	EXCLUDE
**Reason for exclusion:**	
