# Supplementary material for: Association between Cardiovascular Disease Risk Factors and Cardiorespiratory Fitness in Firefighters: A Systematic Review and Meta-Analysis
Source: Int J Environ Res Public Health. 2023 Feb 5;20(4):2816. doi: 10.3390/ijerph20042816 (PMC9957465; doi:10.3390/ijerph20042816)
Supplement: Supplementary file 1 [file ijerph-20-02816-s001.zip › 3. Supplementary File S3_Data Extraction Sheets.pdf]

UNIVERSITY OF THE WESTERN CAPE

Private Bag X 17, Bellville 7535, South Africa

Tel: +27 21-959 2653, Fax: 27 21-959 3686,

E-mail: 3405618@myuwc.ac.za

DATA EXTRACTION SHEETS

General Description

Author	Target Population	Geographical Location	Aim	Problem Statement

UNIVERSITY OF THE WESTERN CAPE

Private Bag X 17, Bellville 7535, South Africa

Tel: +27 21-959 2653, Fax: 27 21-959 3686,

Methodology

Author	Study Design	Theoretical Underpinnings	Sampling Method and Size	Data Collection Methods and Instruments

UNIVERSITY OF THE WESTERN CAPE
Private Bag X 17, Bellville 7535, South Africa
Tel: +27 21-959 2653, Fax: 27 21-959 3686,

Findings and Analysis

Author	Methods of Data Analysis	Study Findings	Author’s Conclusions
