# Supplementary material for: Association between Cardiovascular Disease Risk Factors and Cardiorespiratory Fitness in Firefighters: A Systematic Review and Meta-Analysis
Source: Int J Environ Res Public Health. 2023 Feb 5;20(4):2816. doi: 10.3390/ijerph20042816 (PMC9957465; doi:10.3390/ijerph20042816)
Supplement: Supplementary file 1 [file ijerph-20-02816-s001.zip › 4. Supplementary File S4_critical appraisal of studies.pdf]

Table S1. critical appraisal of cross-sectional studies (adapted from the appraisal tool for cross-sectional studies checklist).

	Espinoza et al. [73]	Atikah et al. [67]	Porto et al. [80]	Barry et al. [49]	Delisle et al. [72]	Houck et al. [76]	McAllister et al. [79]	Nogueira et al. [19]	Li et al. [77]	Donovan et al. [41]	Kiss et al. [75]	Baur et al. [68]	Baur et al. [68]	Strauss et al. [40]	Kirlin et al. [74]	Vicente et al. [84]	Durand et al. [35]	Baur et al. [68]	Perroni et al., [42]	Seyedmehdi et al. [30]	Cameron et al. [71]
**Introduction**																					
Were the aims and objectives clear?	✓	✓	✓	✓	✓	✓	✓	✓	✓	✓	✓	✓	✓	✓	✓	✓	✓	✓	✓	✓	✓
**Methods**																					
Was the study's design in line with the stated goals or objectives?	✓	✓	✓	✓	✓	✓	✓	✓	✓	✓	✓	✓	✓	✓	✓	✓	✓	✓	✓	✓	✓
Was the sample size appropriate?	×	×	×	✓	✓	✓	✓	✓	✓	✓	✓	✓	✓	✓	✓	✓	✓	✓	✓	✓	✓
Was the target demographic adequately defined?	✓	✓	✓	✓	✓	✓	✓	✓	✓	✓	✓	✓	✓	✓	✓	✓	✓	✓	✓	✓	✓
Was the sample frame taken from an appropriate population base to fairly reflect the target population or the reference population?	×	✓	×	×	×	×	×	✓	✓	✓	✓	✓	✓	✓	✓	✓	✓	✓	✓	✓	✓
Was it likely that participants chosen through the selection procedure would represent the target population?	×	×	×	×	×	×	×	✓	✓	✓	✓	✓	✓	×	×	✓	✓	✓	✓	✓	✓
Were precautions taken to address and classify non-responders?	×	✓	✓	×	×	×	×	×	×	✓	×	×	×	✓	✓	×	✓	✓	✓	✓	✓
Were the risk factor and outcome variables assessed applicable to the study's objectives?	✓	✓	✓	✓	✓	✓	✓	✓	✓	×	✓	✓	✓	✓	✓	✓	✓	✓	✓	✓	✓
Instrumental measurements of the risk factor and outcome variables were accurate and reliably used in previous research?	✓	✓	✓	✓	✓	✓	✓	✓	✓	✓	✓	✓	✓	✓	✓	✓	✓	✓	✓	✓	✓
Is it apparent how statistical significance was determined?	✓	✓	✓	✓	✓	✓	✓	✓	✓	✓	✓	✓	✓	✓	✓	✓	✓	✓	✓	✓	✓

Were the procedures sufficiently explained to allow for their replication?	✓	✓	✓	✓	✓	✓	✓	✓	✓	✓	✓	✓	✓	✓	✓	✓	✓	✓	✓	✓	✓
**Results**																					
Were the fundamental data sufficiently explained?	✓	✓	✓	✓	✓	✓	✓	✓	✓	✓	✓	✓	✓	✓	✓	✓	✓	✓	✓	✓	✓
Is non-response bias an issue given the response rate?	✓	✓	✓	✓	✓	✓	✓	✓	✓	✓	✓	✓	✓	✓	✓	✓	✓	✓	✓	✓	✓
Has data pertaining to non-responders been described?	NA	NA	NA	NA	NA	NA	NA	NA	NA	NA	NA	NA	NA	NA	NA	NA	NA	NA	NA	NA	NA
Was there internal consistency in the results?	✓	✓	✓	✓	✓	✓	✓	✓	✓	✓	✓	✓	✓	✓	✓	✓	✓	✓	✓	✓	✓
Were the findings for all the analyses mentioned in the procedures/methods presented?	✓	✓	✓	✓	✓	✓	✓	✓	✓	✓	✓	✓	✓	✓	✓	✓	✓	✓	✓	✓	✓
**Discussion**																					
Were the results sufficient to support the authors' discussions and conclusions?	✓	✓	✓	✓	✓	✓	✓	✓	✓	✓	✓	✓	✓	✓	✓	✓	✓	✓	✓	✓	✓
Were the study's limitations discussed?	✓	×	✓	✓	✓	✓	✓	✓	✓	✓	✓	✓	✓	✓	✓	✓	✓	✓	✓	✓	✓
**Other**																					
Were there any conflicts of interest that could have influenced how the writers interpreted the data?	✓	✓	✓	✓	✓	✓	✓	✓	✓	✓	✓	✓	✓	✓	✓	✓	✓	✓	✓	✓	✓
Did participants give their ethical permission or consent?	✓	✓	✓	✓	✓	✓	✓	✓	✓	✓	✓	✓	✓	✓	✓	✓	✓	✓	✓	✓	✓
FINAL SCORE	15	16	16	16	16	16	17	17	18	18	18	18	18	18	18	18	19	19	19	19	19

Table S2. Critical Appraisal Skills Programme of cohort and case-controlled studies (adapted from the Critical Appraisal Skills Programme).

**CASP – Cohort Study**	Li et al. [77]	Punakallio et al. [82]	Cameron et al. [71]	**CASP – Case Controlled Study**	Vandersmissen et al. [83]
1. Was the study's topic well defined?	1	1	1	1. Was the study's topic clearly defined?	1
2. Was the cohort appropriately recruited?	1	1	1	2. Did the authors approach answering their research question in a suitable way?	1
3. Was the bias minimized by appropriately measuring the exposure?	1	1	1	3. Were the subjects appropriately recruited?	1
4. Was the results correctly measured to reduce bias?	1	1	1	4. Were the controls chosen in a proper manner?	1
5. (A) Have all significant confounding variables been recognized by the authors?	1	1	1	5. Was the bias minimized by appropriately measuring the exposure?	1
5. (B) Have the confounding variables been considered in the design and/or analysis?	1	1	1	6. (A) Were the groups treated identically aside from the experimental intervention?	1
6. (A) Was the participant follow-up thorough enough?	1	1	1	6. (B) Did the authors account for any potential confounding variables in their analysis or design?	1
6. (b) Was the length of the subjects' follow-up sufficient?	1	1	1	7. How significant was the treatment's impact?	NA
7. What were the findings of this research?	1	1	1	8. How accurate was the treatment effect estimate?	1
8. How accurate are the findings?	1	1	1	9. Do you accept the findings?	1
9. Do you accept the findings?	1	1	1	10. Can the local population have the results applied to them?	1
10. Can the local population have the results applied to them?	1	1	1	11. Do the findings of this study accord with other pieces of information?	1
11. Do the findings of this study accord with other pieces of information?	1	1	1		

12. What practical implications does this study have?	1	1	1		
FINAL SCORE	14	14	14	FINAL SCORE	11

Note: NA – indicates not applicable; ✓ – indicates yes.
