# Supplementary material for: Association between Cardiovascular Disease Risk Factors and Cardiorespiratory Fitness in Firefighters: A Systematic Review and Meta-Analysis
Source: Int J Environ Res Public Health. 2023 Feb 5;20(4):2816. doi: 10.3390/ijerph20042816 (PMC9957465; doi:10.3390/ijerph20042816)
Supplement: Supplementary file 1 [file ijerph-20-02816-s001.zip › 5. Supplementary File S5_Forest Plot and Eggers test for publication Bias.pdf]

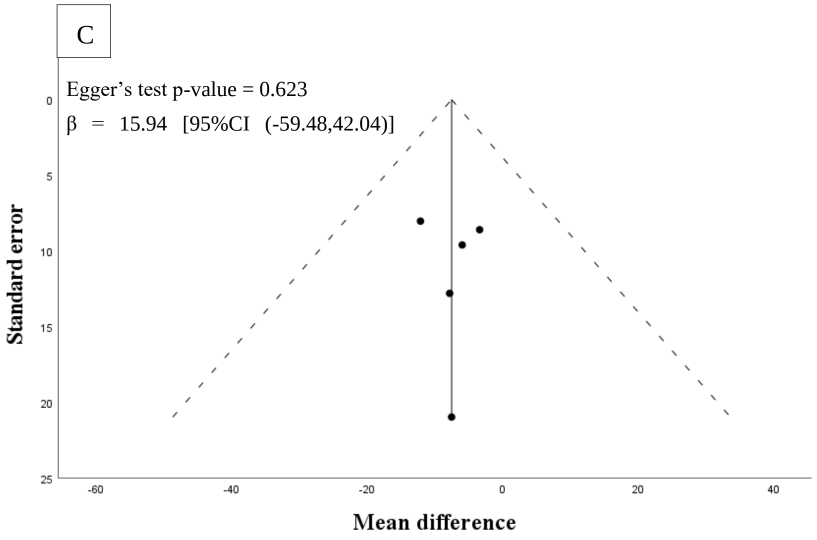

Figure S2C: Funnel plot for publication bias.

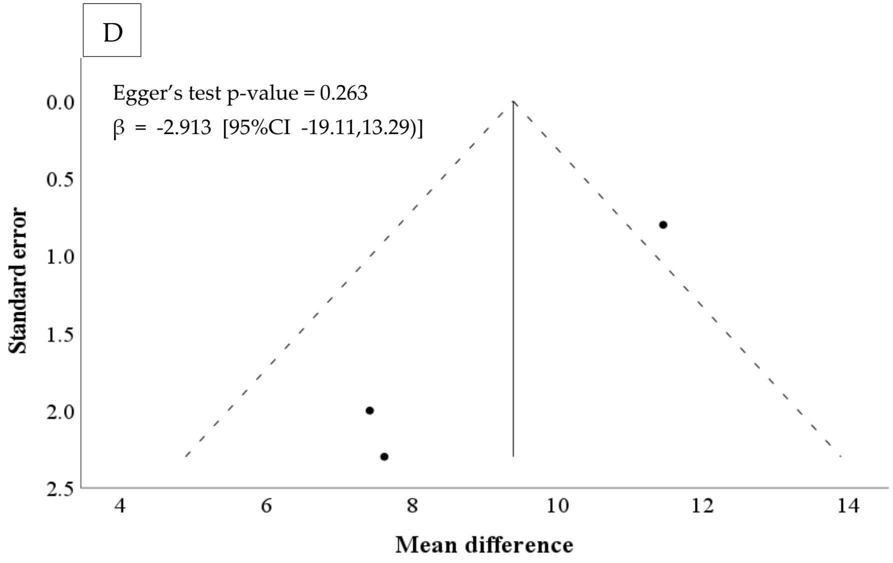

Figure S2D: Funnel plot for publication bias.

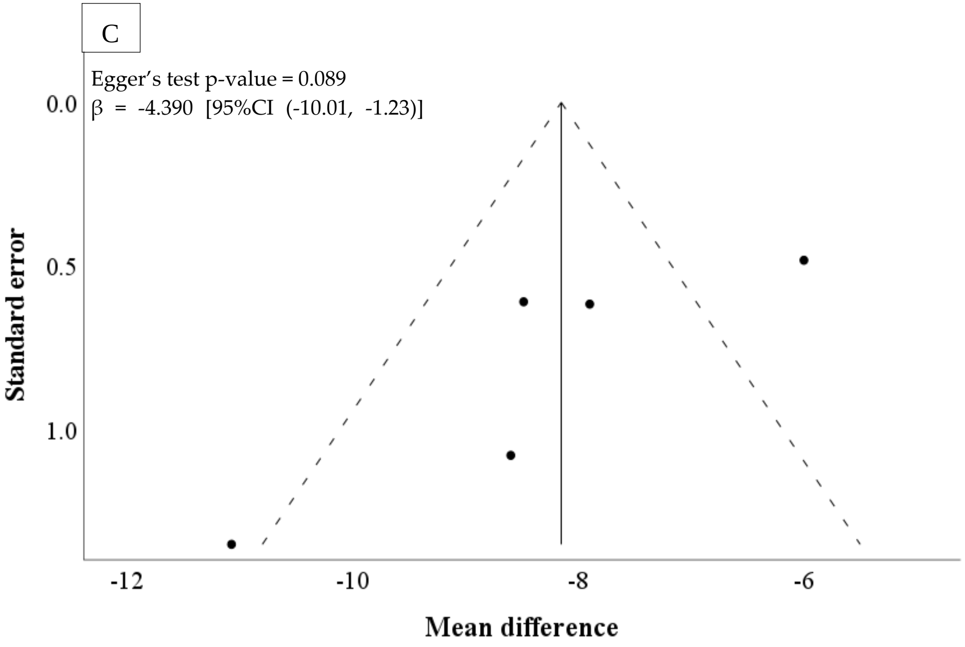

Figure S3C: Forest plot for publication bias.

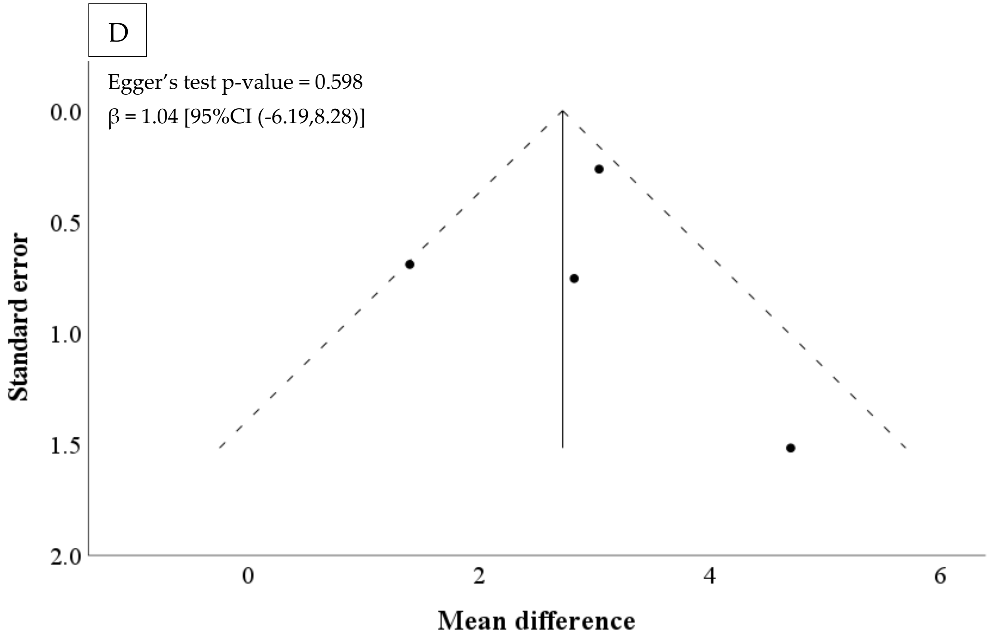

Figure S3D: Forest plot for publication bias.

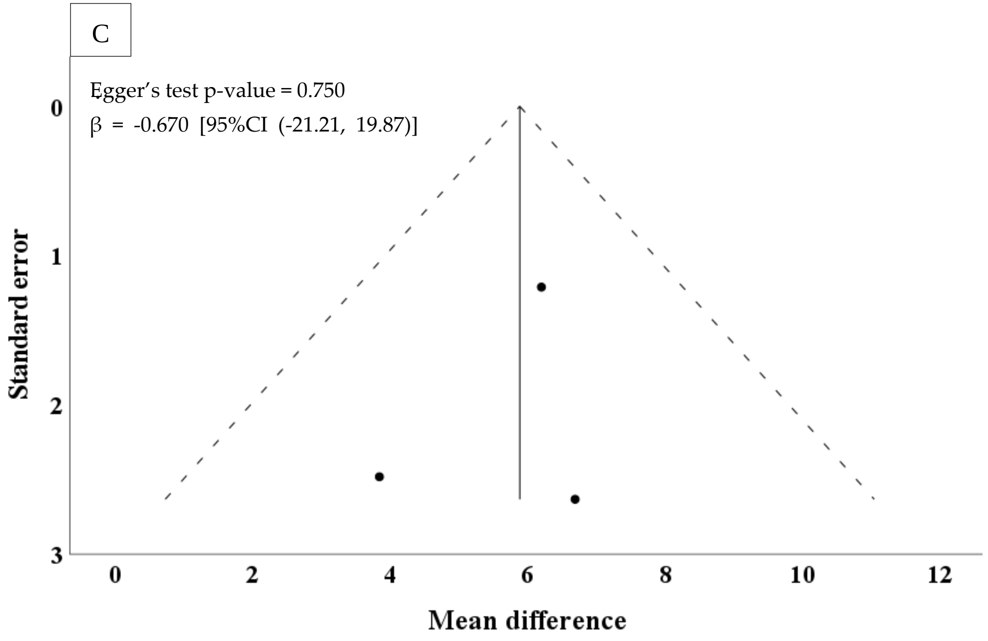

Figure S4C: Funnel Plot for publication bias.

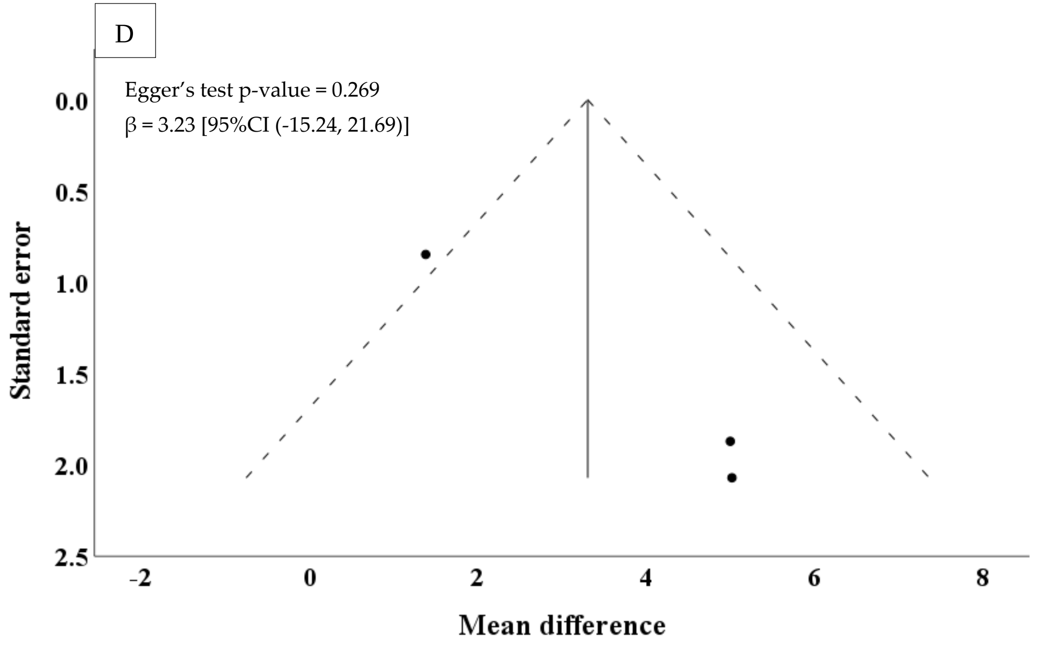

Figure S4D: Funnel plot for publication bias.

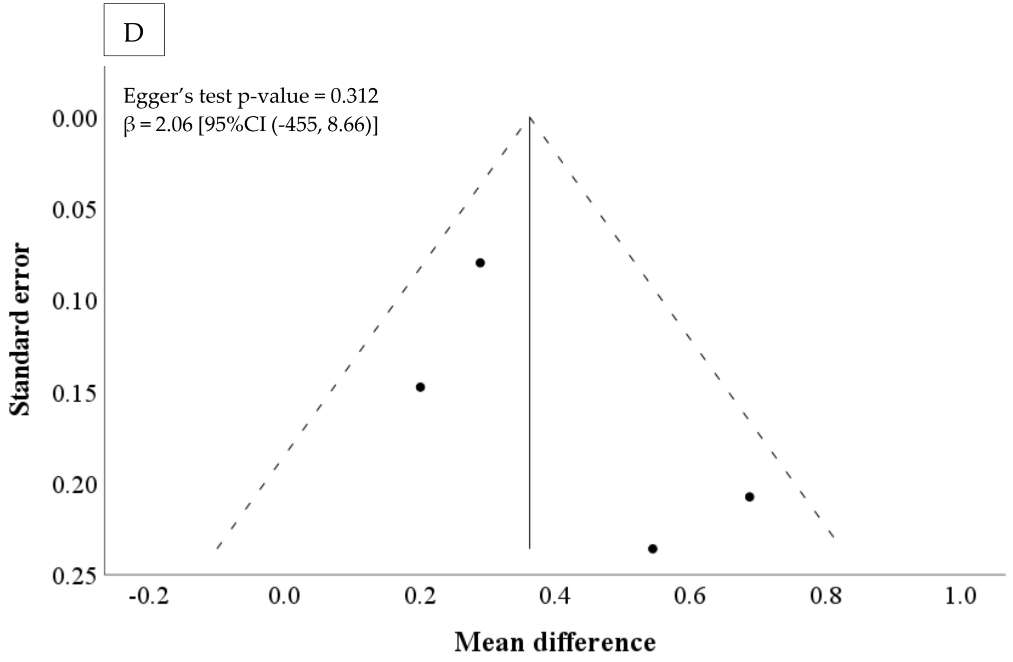

Figure S5D: Funnel plot for publication bias.

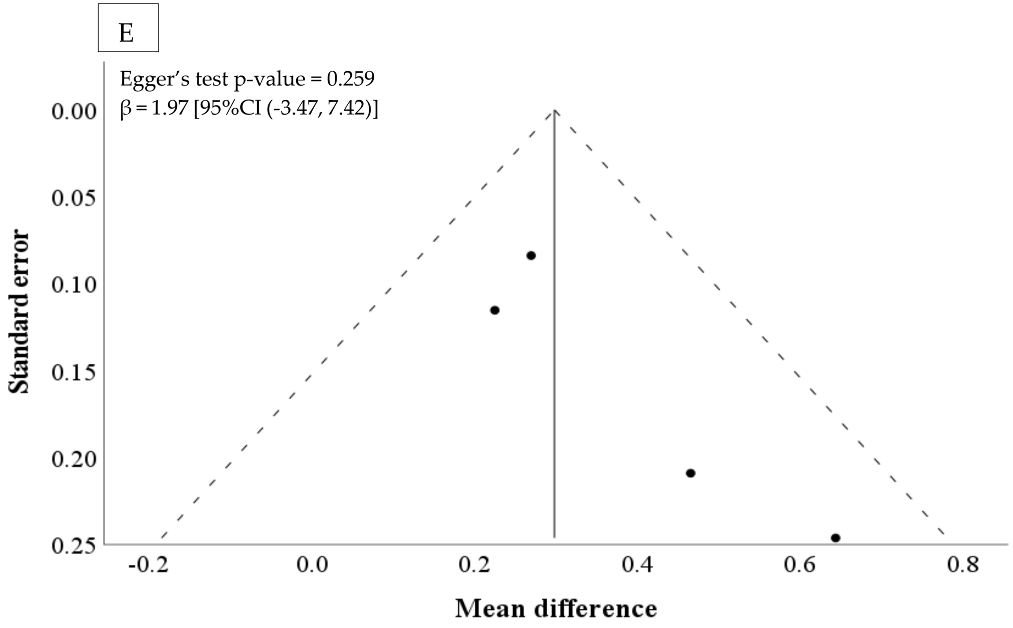

Figure S5E: Funnel plot for publication bias.

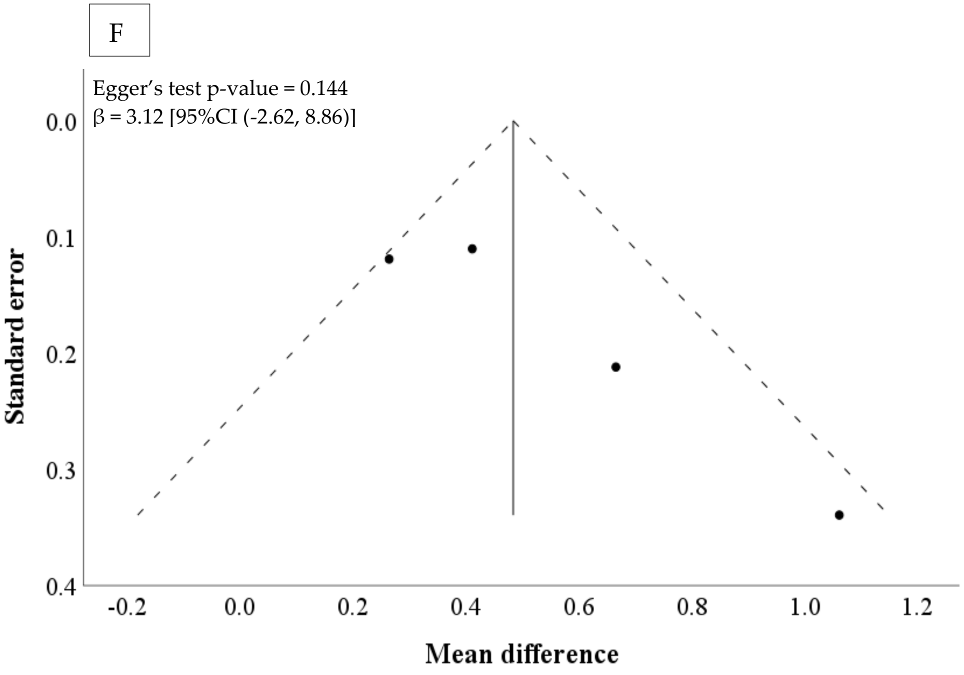

Figure S5F: Funnel plot for publication bias.

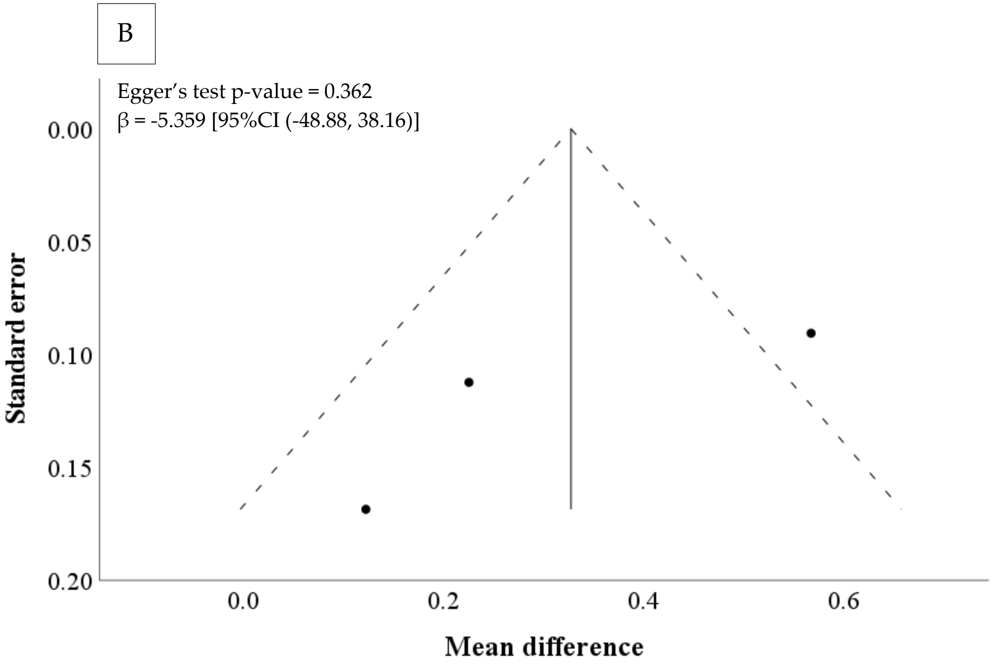

Figure S6B: Funnel plot for publication bias.
